# Machine Learning-Based Virtual Screening for the Identification of Cdk5 Inhibitors

**DOI:** 10.3390/ijms231810653

**Published:** 2022-09-13

**Authors:** Miriana Di Stefano, Salvatore Galati, Gabriella Ortore, Isabella Caligiuri, Flavio Rizzolio, Costanza Ceni, Simone Bertini, Giulia Bononi, Carlotta Granchi, Marco Macchia, Giulio Poli, Tiziano Tuccinardi

**Affiliations:** 1 Department of Pharmacy, University of Pisa, Via Bonanno 6, 56126 Pisa, Italy; 2 Department of Life Sciences, University of Siena, Via Aldo Moro, 2, 53100 Siena, Italy; 3 Pathology Unit, Centro di Riferimento Oncologico di Aviano (CRO) IRCCS, 33081 Aviano, Italy; 4 Department of Molecular Sciences and Nanosystems, Ca’ Foscari University, 30123 Venezia, Italy

**Keywords:** virtual screening, machine learning, kinase, CDK5

## Abstract

Cyclin-dependent kinase 5 (Cdk5) is an atypical proline-directed serine/threonine protein kinase well-characterized for its role in the central nervous system rather than in the cell cycle. Indeed, its dysregulation has been strongly implicated in the progression of synaptic dysfunction and neurodegenerative diseases, such as Alzheimer’s disease (AD) and Parkinson’s disease (PD), and also in the development and progression of a variety of cancers. For this reason, Cdk5 is considered as a promising target for drug design, and the discovery of novel small-molecule Cdk5 inhibitors is of great interest in the medicinal chemistry field. In this context, we employed a machine learning-based virtual screening protocol with subsequent molecular docking, molecular dynamics simulations and binding free energy evaluations. Our virtual screening studies resulted in the identification of two novel Cdk5 inhibitors, highlighting an experimental hit rate of 50% and thus validating the reliability of the in silico workflow. Both identified ligands, compounds **CPD1** and **CPD4**, showed a promising enzyme inhibitory activity and **CPD1** also demonstrated a remarkable antiproliferative activity in ovarian and colon cancer cells. These ligands represent a valuable starting point for structure-based hit-optimization studies aimed at identifying new potent Cdk5 inhibitors.

## 1. Introduction

Cyclin-dependent kinases (Cdks) are serine/threonine protein kinases that are primarily involved in the regulation of cell cycle, cell differentiation and gene transcription [1]. The Cdk family consists of 20 serine/threonine protein kinases, including Cdk5 that often have tissue-dependent functions. Cdk5 is considered as an atypical member of the mammalian Cdk family. Although other Cdks are primarily found in proliferating cells, Cdk5 is predominantly located in postmitotic neurons [2], where it plays a vital role in brain development, neuronal survival, synaptic plasticity, microtubule regulation and pain signaling [3]. Unlike other Cdks, Cdk5 is activated by the non-cyclin proteins, p35 and p39, and their truncated products, p25 and p29, which are abundantly expressed in the brain. The dysregulation of Cdk5 can mainly be attributed to its activation by p25, which results in the hyperactivation of this Cdk and its aberrant subcellular localization, with dysfunctional consequences at the neuronal level [4,5]. In fact, Cdk5 is implicated in neurodegenerative diseases such as Alzheimer’s disease (AD) and Parkinson disease (PD) by contributing to the formation of the aberrantly phosphorylated tau protein [5] and to the degeneration of dopaminergic neurons in the substantia nigra [6]. Furthermore, marked Cdk5 immunoreactivity was observed in degenerating neurons in the spinal cord of patients with sporadic Amyotrophic lateral sclerosis (ALS) [7] as well as in brain samples from patients with HIV encephalitis, which exhibit more profound cognitive alterations and neurodegeneration [8,9]. Recent reports also highlight the link between Cdk5 hyperactivation and diabetes-associated neurodegeneration; the high-glucose exposure leads to p25 generation and protein tau hyperphosphorylation, determining neuronal death and cognitive impairment [10]. Although its altered regulation mainly leads to neurodegeneration, Cdk5 is ubiquitous, and its aberrant expression has been observed in multiple types of solid and hematological malignancies [11]. In fact, an increased expression of Cdk5 have been reported in pancreatic [12], colorectal [13], prostate [14], breast [15] and ovarian [16] cancers, glioblastoma multiforme [17] and multiple myeloma [18]. Indeed, the impacts of Cdk5 on the hallmarks of cancer are different, such as the uncontrolled proliferative signaling and its aftermath: growth suppression [19], tumour promoting inflammation [20], invasion and metastasis [21], induction of angiogenesis [22], genomic instability and mutation [23]. This multifaceted role in causing diseases makes Cdk5 a promising drug target [24]. Roscovitine (seliciclib) and dinaciclib are currently the prototype inhibitors in clinical trials that target multiple kinases, including Cdk5 [25,26,27]. Several phase I and phase II clinical trials in cancer patients have been completed for these inhibitors and a recent Phase III clinical trial demonstrated the potential antiproliferative activity of dinaciclib [28]. Nevertheless, the search for Cdk5 inhibitors as drug candidates endowed with an optimal potency and selectivity continues, given the current unavailability of specific Cdk5 inhibitors at any stage of drug development.

Herein, we report machine learning-based Virtual Screening (VS), followed by docking evaluations and molecular dynamics (MD) simulations, carried out with the aim of discovering novel potential Cdk5 inhibitors. Indeed, machine learning (ML) proved to be a promising strategy for the identification of drug leads through VS, since it allowed the discovery of two novel compounds endowed with micromolar inhibitory activity against Cdk5, thus validating the reliability of our predictive ML models. Furthermore, the availability of X-ray structures of Cdk5 in complex with reference inhibitors allowed the employment of docking protocols for predicting the ligand-binding dispositions, which were further studied through MD simulations and binding free energy evaluations, thus providing a starting point for future structure-based hit optimization studies aimed at designing new and more potent Cdk5 inhibitors.

## 2. Results and Discussion

### 2.1. Machine Learning Model Generation, Optimization and Evaluation

In order to develop ML models for identifying novel ligands potentially active against Cdk5, we searched for compounds with bioactivity data related to Cdk5 inhibition available on ChEMBL 25. Compounds with biological activity measured as *K*_i_ were retrieved and subjected to a data curation process, eventually obtaining the training set used to generate our ML models. The test set was assembled by employing compounds with biological activity measured as IC_50_ available on ChEMBL 25. The final test set was also enriched with compounds removed from the training set (see Materials and Methods for details). Training set and test set instances were labeled as active if their potency was ≤1 µM and inactive when their potency was ≥5 µM. Compounds with potency between the two thresholds were removed. The final training set consisted of 196 active and 462 inactive compounds, whereas the test set included 98 compounds labeled as active and 216 as inactive. The generated models were obtained by representing the training set compounds through molecular fingerprints (FPs). Six different types of FPs were used: Morgan, RDKit, Layered, Pattern, MACCS and Pharm2D. Each FP method was combined with four different classification algorithms, i.e., Random Forest (RF), Support Vector Machine (SVM), k-Nearest Neighbor (KNN) and Multi-Layer Perceptron (MLP), for the development of a total of 24 different classification models. Each model combination was subjected to an optimization process in order to identify the best hyperparameter configuration for maximizing its performance. A 10-folds cross-validation (CV) analysis was then performed for each optimized model to obtain an overview of its predictive capacity. The performance of all models was evaluated and ranked according to the values of Matthew’s Correction Coefficient (MCC) obtained through CV analysis (see Materials and Methods for details). The results obtained from the CV provided insights into the impact of the different FPs on the quality of predictions. In particular, we observed that for each algorithm, Morgan FPs allowed to obtain the highest predictive performance. Indeed, Morgan FPs showed a minimum MCC value of 0.60, as in the case of KNN-based models, and a maximum of 0.69, as seen in SVM-based models. On the contrary, the FPs that proved to be the worst in terms of global performance were MACCS FPs, with MCC values ranging from 0.47 to 0.55 (Tables S1–S4 in Supporting Information). These results highlight that Morgan FPs were able to provide the best molecular description to generate patterns for the discrimination of Cdk5 inhibitors from inactive molecules. According to these findings, models based on each ML algorithm and Morgan FPs were then subjected to a final external evaluation consisting of predicting the activity of the test set compounds. For each selected model, the performance of the test set prediction was evaluated considering Precision and Recall, besides MCC (as reported in Materials and Methods). Indeed, the success of a VS protocol is defined by its ability to maximize true positive predictions over false positive ones (Precision), rather than maximizing the identification of true positives over the total number of positives in the dataset (Recall). The importance of improving Precision is related to the extremely small number of compounds typically selected for biological evaluation, in contrast to the larger number of compounds present in the screened library, and thus to the need of maximizing the VS hit rate. On the other hand, improving the ability of the model to retain true positives, while reducing the number of false positives, is often associated with an inevitable increase of active compounds misclassified as false negatives. This well-known issue is crucial for the selection of the appropriate metric to evaluate the VS protocol performance. In this case, a metric such as F1, which corresponds to the harmonic mean of Precision and Recall, is not appropriate since a high F1 value can often be biased by a high Recall, thereby masking a low Precision [29]. Based on these considerations, in this study, we focused on Precision score for the selection of the best model to be applied for VS, among those showing the highest MCC values. The results obtained from the external evaluation (Table 1) show that all selected models except KNN performed equally well in terms of MCC; in fact, KNN showed an MCC value (0.33) significantly lower than those obtained for the other models (0.41–0.43). In terms of precision, RF achieved a score of 0.87 with a margin of 0.22 over the second best model (SVM). An opposite trend can be observed when considering Recall, since RF obtained the lowest score, whereas MLP appeared to be the best one, with a score of 0.61. With the aim to improve the predictive performance achievable through the use of our models, we employed a consensus strategy that allowed us to combine the different predictions of the four top-scored models selected. In fact, we already successfully applied consensus strategies for improving the reliability of docking studies [30], developing efficient VS protocols [31], and also boosting the predictive performance of our ML-based platform for small-molecule toxicity predictions [32]. In this context, a compound was classified as active only if predicted as active by all four models, i.e., if the Probability Score (PS) provided by each model was at least 0.5 (see Materials and Methods for details). The consensus approach was evaluated through to the prediction of the test set by measuring MCC, Precision and Recall in comparison with the values obtained for each individual model. Table 1 shows that the consensus approach performed slightly better than the single best model (RF) in terms of MCC and Precision, achieving only a small improvement of 0.01; this was also observed for Recall, which thus maintained a value (0.31) far below that observed for MLP (0.61), the best algorithm in terms of this specific evaluation metric.

**Figure 1:**
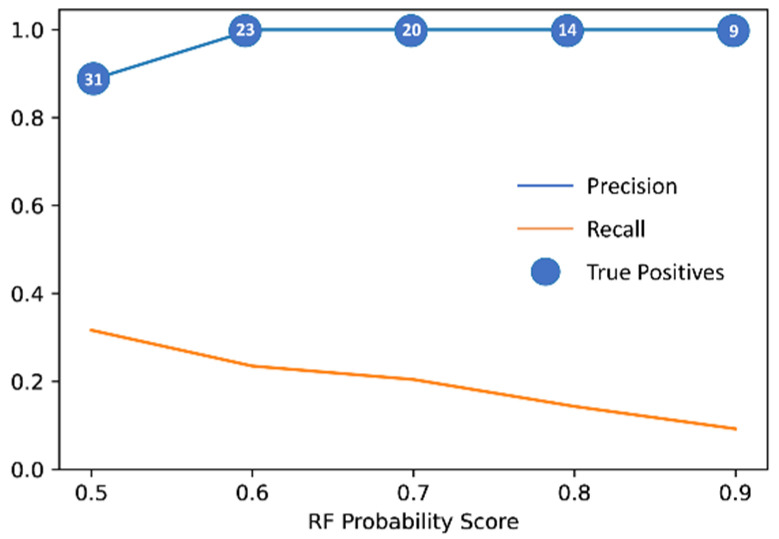
Performance evaluation of RF model with different classification thresholds, based on test set prediction. The test set consists of 314 instances of which 98 active.

Given the great importance of Precision for VS studies, we decided to focus on the RF model and the consensus approach. However, although the consensus showed to be the best approach, the only slight improvement over RF in terms of MCC and Precision, compared to the greater amount of time required for generating multiple predictions versus a single one, led us to choose RF as the final model for the VS step. In order to minimize the possibility of predicting false positives in the VS, the performance evaluation of the model based on the test set was repeated by changing the prediction criteria. Specifically, under normal conditions, a compound is predicted to be active if the PS provided by the model is at least 0.5; our goal was to study how the performance of the model could be affected by increasing the PS threshold by steps of 0.1, up to the maximum value of 0.9. This analysis allowed us to evaluate the impact of the confidence of the predictions on the predictive performance of the model, trying to find an optimal classification threshold for maximizing Precision (Figure 1).

According to our expectation, the observed trend showed an increase in Precision at the expense of Recall. In other words, increasing the PS threshold for prediction resulted in fewer false positives but, on the other hand, in an increased number of false negatives. This phenomenon is due to the fact that most active compounds present a PS that is not particularly high; therefore, they tend to be classified as inactive when using threshold values greater than 0.5.

Looking at Figure 1, it can be observed that after the first threshold increase, the Precision score achieves the maximum possible value, which is maintained with the other thresholds. However, according to what discussed above, by increasing the PS threshold the number of true positive predictions is reduced. Indeed, the RF model was still able to predict 23 true positives (out of a total of 91 active instances) with the threshold of 0.6, whereas only 9 true positives were identified when 0.9 was considered as the classification threshold. These findings show that using a classification threshold of at least 0.6 maximizes the Precision of the model, thus providing an important indication for identifying new active compounds through VS studies.

### 2.2. Virtual Screening

In light of the results of the previous analyses, a practical application of the developed model was performed through a VS study. Two focused libraries of commercial compounds, belonging from Enamine and Vitas-M (see Material and Methods for details), were subjected the same data curation protocol used for training and test set compounds, in order to have consistent molecular representations, and independently screened using the optimized RF model. A total of 548 compounds within the Enamine library were associated to a PS of at least 0.5, whereas the highest PS corresponding to 0.8 was obtained for only four compounds. Conversely, only four compounds within Vitas-M library were predicted as active, with a maximum probability score of 0.5. Based on the results obtained in the performance analysis of the model, we decided to retain only the compounds with the highest PS for each library, in order to select a reasonable number of compounds to be purchased and subjected to biological evaluations with the highest chance of being actually active. Therefore, the VS study led to the selection of eight total compounds, four from each commercial library considered. The eight selected compounds were subjected to clustering and visual inspection, and only four structurally different compounds were finally purchased and tested for Cdk5 inhibitory activity. Enzymatic inhibition assays revealed that only two out of the four compounds, namely **CPD1** and **CPD4**, were endowed with promising Cdk5 inhibitory activity, with IC_50_ values of 3.43 and 1.27 µM, respectively, corresponding to a hit ratio of 50% (Table 2 and Figure S1).

### 2.3. Antiproliferative Assays

Compounds CPD1 and CPD4 were also selected for further in vitro experiments to evaluate their antiproliferative potency against cancer cells. Cisplatin was used as the reference compound. Due to the key role that Cdk5 plays in the tumor progression of breast, colorectal and ovarian cancers [13,16,33], four tumor cell lines were chosen: the human breast MDA-MB-231, the colorectal HCT116 and the ovarian OVCAR3 and A2780 cancer cells (Table 3). Compounds CPD1 and CPD4 produced an appreciable inhibition of cell viability in all the tested cell lines, with IC_50_ values ranging from 12 to 2127 nM. In particular, compound CPD1 showed a more potent cytotoxic activity on OVCAR3 and A2780, with IC_50_ values of 12.0 and 93.7 nM (IC_50_ = 681 and 275 nM, respectively, for cisplatin). The proliferation of the HCT116 tumor cells was similarly affected by CPD4 and cisplatin, whereas CPD1 was more potent (about 10-fold). Finally, compounds CPD1 and CPD4 exerted a lower antiproliferative potency on MDA-MB-231, always maintaining better activities than those of the reference inhibitor (IC_50_ ranging from 1474 to 2127 nM for the two compounds versus > 10,000 nM of cisplatin).

### 2.4. Molecular Modeling Studies

**Figure 2:**
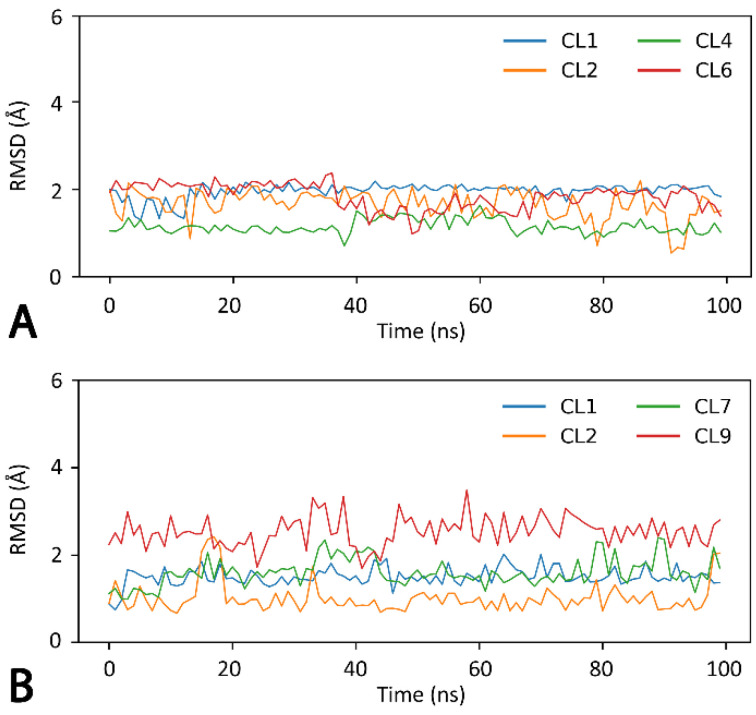
Analysis of the MD simulations of the different Cdk5-CPD1 (**A**) and Cdk5-CPD4 (**B**) complexes.

With the aim of providing a hypothetical binding orientation of CPD1 and CPD4 inside Cdk5 binding site, molecular docking, MD simulations and binding free energy evaluations were performed by using the X-ray structure of Cdk5 in complex with a naphtyridine inhibitor (PDB code 7VDR) [34]. CPD1 and CPD4 were subjected to a robust docking procedure with GOLD software by using the PLP scoring function. The docking results were clustered within a threshold of 2.0 Å and the representative binding modes were filtered based on cluster population and presence of H-bonds with the protein hinge region (see the Material and Methods for details). A total of four potentially reliable binding conformations were thus identified for both CPD1 and CPD4. Each binding mode was then analyzed through a 100 ns MD simulations protocol aimed at evaluating the stability of the predicted binding disposition of the ligands and their key interactions with the protein. The root-mean square deviation (RMSD) of the ligand’s position during the MD, with respect to the original docking pose, was thus analyzed for each studied ligand–protein complex. The analysis showed high stability of all binding modes predicted for CPD1, with an average RMSD around or below 2 Å (Figure 2A). Similar results were obtained for CPD4, whose RMSD values did not exceed an average of 3 Å, although cluster 9 presented larger fluctuations (Figure 2B).

Given the stability of all selected poses for both CPD1 and CPD4, we decided to consider all of them for further analyses. To better assess the reliability of the different Cdk5-ligand complexes, the corresponding ligand–protein binding free energies were evaluated from the MD coordinates extracted from the last 50 ns of simulation. The Molecular Mechanics-Poisson Boltzmann surface area (MM-PBSA) method was used for the calculation (see Material and Methods for details). This approach analyzes the MD simulation snapshots and calculates the contributions of both gas-phase and solvation free energies for the unbound ligand, unbound protein and bound complex [35]. As shown in Table 4, the analysis identified cluster 1 as the most reliable binding mode for CPD1, since it showed the best binding energy (ΔPBSA = −35.9 kcal/mol) and exceeded by 2.8 kcal/mol the interaction energies associated with the second best pose. However, given the small energy window between clusters 2 and 1, we decided to consider both of them in subsequent binding mode analyses. In support of our decision, the energy gap between cluster 2 and the third ranked pose was 7.1 kcal/mol, highlighting the lower stability of the other poses. For CPD4, the results shown in Table 4 highlighted cluster 7 (ΔPBSA = −34.4 kcal/mol) as the most energetically favored binding mode, with a difference of 5.0 kcal/mol from the second ranked pose.

**Figure 3:**
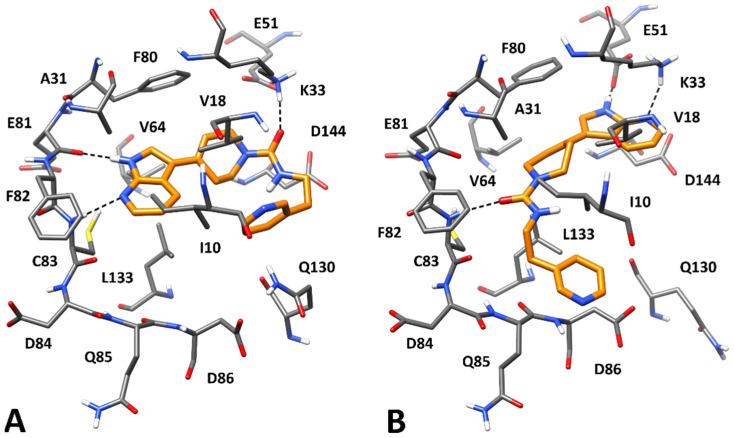
Minimized average structures of CPD1 (orange) in complex with Cdk5. The two different binding modes represented by (**A**) cluster 1 and (**B**) cluster 2 are shown. The protein residues surrounding the ligand, constituting the binding site, are shown as grey sticks, whereas hydrogen bonds are shown as black dashed lines.

Based on the results obtained from the MM-PBSA analysis, two binding dispositions for CPD1 (clusters 1 and 2) and one for CPD4 (cluster 7) were selected for a visual analysis. Figure 3 shows the binding modes refined through the MD simulations predicted for CPD1. Based on the most energetically favorable binding disposition of CPD1, represented by cluster 1, the ligand places its azaindole core within the hinge region of the protein, forming two different H-bonds with the backbone nitrogen of C83 and the backbone oxygen of E81 through its pyridine nitrogen and pyrrole NH, respectively. Moreover, the bicyclic core forms hydrophobic interactions with V18, A31, V46, F80 and, in particular, with L133, over which it perfectly lays. The carbonyl group of CPD1 establishes an additional H-bond with K33, which further stabilizes the binding mode of the ligand. Finally, the piperidine moiety of CPD1 shows lipophilic interactions mainly with V18 and F80, whereas the pyridine ring forms an NH-π interaction with the side chain of Q130 and takes hydrophobic contacts with I10 and V18; all these interactions appear to stabilize the bent conformation of the terminal part of the ligand. In the binding orientation represented by cluster 2 (Figure 3B), CPD1 forms only an H-bond with the amide backbone of C83, within the hinge region of Cdk5, through its central carbonyl group. In fact, the azaindole core of the ligand is located outside the hinge region of the protein and participates in a double H-bond interaction with K33 and E51. Specifically, the pyridine nitrogen acts as an H-bond acceptor with K33, whereas the pyrrole NH group acts as an H-bond donor with E51. Moreover, the bicyclic core of the ligand forms hydrophobic interactions mainly with F80, whereas the piperidine ring shows lipophilic interactions with V18, A31, V64 and L133. Finally, the terminal pyridine moiety of CPD1 is located in the solvent-exposed region of the binding site, forming van der Waals interactions only with I10 and D86. Although both binding modes predicted for CPD1 appear as reliable, since most kinase inhibitors present an H-bond donor/acceptor moiety that interact with the hinge region residues, the binding mode of CPD1 represented by cluster 1 is believed to be more reliable than the binding disposition represented by cluster 2. This is also in agreement with the binding free energy values obtained for the two binding dispositions of CPD1, suggesting cluster 1 as the most energetically favored one.

**Figure 4:**
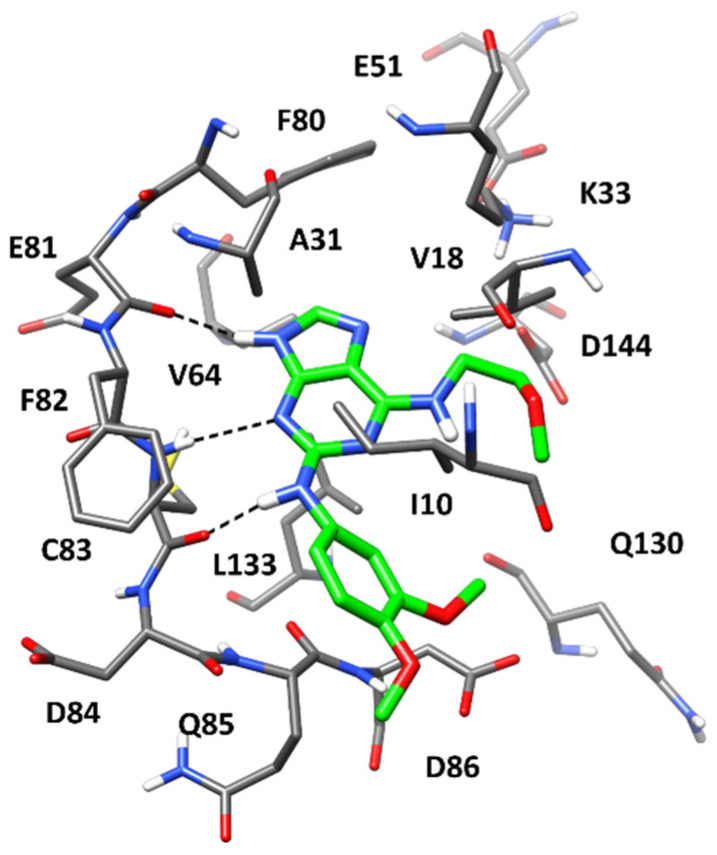
Minimized average structures of CPD4 (green) in complex with Cdk5. The protein residues surrounding the ligand, constituting the binding site, are shown as grey sticks, whereas hydrogen bonds are shown as black dashed lines.

Figure 4 shows the predicted binding mode for CPD4 derived from cluster 7. A key interaction network between the purine core of the ligand and the hinge region of Cdk5 can be observed. In particular, the binding mode of the ligand is characterized by the presence of two H-bonds with both the backbone oxygen and nitrogen of C83 and a further H-bond with the backbone oxygen of E81.

Moreover, the bicyclic core of the ligand forms hydrophobic interactions mainly with A31 and L133, as well as with V46 and F80. The dimethoxybenzene ring is oriented toward the solvent-exposed region of the binding site, sandwiched between Q85 and D86 from one side, and I10 from the other, thus forming interactions with these residues. Finally, the methoxyethyl tail of CPD4 mainly forms hydrophobic interactions with the side chain of V18.

## 3. Materials and Methods

### 3.1. Machine Learning Data Sets

Our primary data source for creating the models was ChEMBL 25 [36]. We collected all compounds with inhibitory potencies against Cdk5 (corresponding to UniProt ID “Q00535”) measured as *K*_i_. A structure refinement protocol was performed using OpenEye chemistry toolkit [37], including charge neutralization, salts removal and control of structural integrity. In order to obtain a unique dataset of compounds, if three or more *K*_i_ values were available for a compound, values deviating by more than 25% from the respective calculated mean were discarded, and the mean *K*_i_ value was recalculated and assigned as the final potency annotation. In the case of two *K*_i_ values, the mean value was used. These steps allowed for obtaining 1019 unique compounds with an unambiguously defined activity. From this dataset, only the molecules tested by Metz and co-workers [38], which almost constituted the entire dataset, were retrieved, whereas only 10 compounds belonging to diverse works were removed, obtaining a dataset with 1009 compounds. This additional step was carried out to ensure the homogeneousness of the dataset in terms of activities, considering potency values measured using the same assay conditions. Based on the distribution of *K*_i_ values associated to the dataset molecules, two thresholds for classifying compounds were defined. Specifically, compounds with *K*_i_ ≤ 1 µM (p*K*_i_ ≥ 6) were labelled as active, whereas compounds with *K*_i_ ≥ 5 µM (p*K*_i_ ≤ 5.3) were categorized as inactive. Compounds with potency between the two thresholds were removed. The resulting refined dataset consisted of 225 compounds labeled as active and 533 inactive compounds. In order to enrich the external test set (see below), 100 random compounds were removed. Therefore, this workflow resulted in a final training set of 658 compounds with 196 active and 462 inactive instances. For the validation of our ML models, we assembled an external test set by recovering from ChEMBL 25 all records of Cdk5 inhibitors with potencies measured as IC_50_ values, for a total of 204 molecules, and then removing compounds already present in the training set. Furthermore, the 10 compounds with *K*_i_ potency values not determined by Metz and co-workers [38], and the 100 random compounds previously excluded from the training set, were included in this external dataset. The classification of the external test set was performed using the same thresholds defined for the training set. The test set resulted in 314 compounds, of which 98 labeled as active and 216 as inactive.

### 3.2. Molecular Representations

The SMILES (Simplified Molecular-Input Line-Entry System) strings of training and test set compounds downloaded from CHEMBL 25 were used to compute different types of molecular fingerprints (FPs), in order to provide the input data for ML algorithms. In this context, we represented both molecular structures and properties using different FPs calculated with the RDKit package [39].

Morgan FPs [40], better known as circular fingerprints, are based on Morgan’s algorithm. These FPs represent the structure of the compounds by computing for each atom its surroundings environment, including atomic bonds within a certain distance or radius. Each atom identifier is then folded into a vector with fixed length through hashing functions provided by RDKit. For this work, the atomic radius was set to two, whereas the vector length was fixed to 2048.

RDKit FPs are RDKit-specific FPs [39]. The algorithm identifies all subgraphs in the molecules within a particular range of atom sizes. For each identified subgraph, a raw bit ID is computed and then the hashing function is fit in the assigned FP size by setting the corresponding bit. Atom types are set by atomic numbers and aromaticity, whereas bond types are set by atom types and bond types. In this work, the length of the FP vector was 2048 bits for consistency with Morgan FPs.

Layered FPs represent other “RDKit original” FPs and were developed with the intention of using them as substructure FPs [39]. Layered FPs employ the same subgraph enumeration algorithm used in RDKit FPs; however, after subgraphs are generated, they are used to set multiple bits based on different atom and bond type definitions.

Pattern FPs were designed to be used in substructure screenings [39]. The algorithm identifies features in the molecule by doing substructure searches using a small number of very generic Smiles Arbitrary Target Specification (SMARTS, a language used for describing molecular patterns and properties) and then hashing each occurrence of a pattern based on the atom and bond types involved.

MACCS FPs (Molecular ACCess System) [41] can be considered the prototype of key-based fingerprints, where the bits are set according to the presence or absence of predefined substructures (structural keys). MACCS keys were developed by MDL (Molecular Design Limited, now a subsidiary of BIOVIA) and there are two sets of keys: a public set, which contains 166 keys and is almost universally implemented in chemioinformatics applications, and a wider set that contains 960 keys and is available only in BIOVIA’s Discovery Studio. For this work, we used MACCS fingerprints computed with the 166 public keys.

Pharm2D FPs are 2D pharmacophore fingerprints created combining a set of chemical features with the 2D (topological) distances between them [39]. When the distances are binned, unique integer IDs can be assigned to each of these pharmacophores, and they can be stored in a fingerprint. For this work, pharmacophore fingerprints were computed considering all possible combinations of default features included within the RDKit library. Specifically, the following feature types were considered: H-bond acceptor, H-bond donor, positive ionizable, negative ionizable and aromatic features. Each feature combination included a minimum of two and a maximum of three features.

### 3.3. Machine Learning Methods

Binary classification models were generated using four different ML algorithms: Random Forest, Support Vector Machine, k-Nearest Neighbors and Multi-layer Perceptron. All ML methods were implemented and tuned using the Scikit-learn python library [42].

Random Forest (RF) is one of the most widely used ensemble ML algorithms that can be used for either classification or regression [43,44]. Each individual tree is trained on a bootstrap sample (sampling with replacement) and the predictions are made by the majority vote of the trees. The main hyperparameters optimized during model building were *max_features*, which indicates the maximum number of features that can be considered in a single tree, and *n_estimators*, expressing the number of trees built before making the averages of predictions. The *number of estimators* that were taken into account corresponds to 100 and 500, whereas the options of *max_features* investigated were: (a) *log2*, which corresponds to the binary logarithm of the total features for a single node; (b) *sqrt*, which is the square root of the total features in a single node; (c) *None*, for which *max_features* correspond to the total number of features.

Support Vector Machine (SVM) maps the data according to their common patterns and aims towards their optimal division between two classes, with each of them entirely lying on opposite sides of a separating hyperplane [45]. In the case of binary classification, the algorithm attempts to construct a hyperplane *H* by maximizing the distance between the training instances belonging to different classes. To control the magnitude of allowable training errors, the regularization hyperparameter *C* is used to balance the size of the hyperplane margins and classification errors, whereas the hyperparameter *kernel* is used to map the data into a higher dimensional feature space in order to make them separable. For hyperparameter *C,* the values 0.01, 0.1, 1, 10 and 100 were considered, whereas two *kernels* were tested during the tuning process: linear and tanimoto [46].

k-Nearest Neighbors (KNN) algorithm is a type of instance-based learning, which computes the distance between the query point and the training instances to determine the k closest points [47]. The final prediction is therefore obtained by the most frequent outcome among the features of the nearest neighbors to the input data. The hyperparameters optimized during model building were *n_neighbors* and *weight* since they both reduce the error due to the voting of the surrounding neighbors. *n_neighbors* represent the number of neighbors taken into consideration for the classification, whereas *weight* indicates how much the different surrounding elements influence the prediction. In this work, two options were tested for *weight*: (a) uniform, indicating that all points in each neighborhood are weighted equally, and (b) distance, imposing that closer neighbors of a query point have a greater influence than neighbors that are further away from it. The values investigated for *n_neighbors* were in a range between 1 and 30.

Multi-layer Perceptron (MLP) is a type of feedforward artificial neural network, that consists of at least three layers: an input layer, a hidden layer and an output layer, each consisting of a set of neurons [48]. Specifically, in the input layer, the number of neurons is set to the number of features for a record in the training data. The neurons contained in each hidden layer process the weighted inputs received from the neurons of the previous layer, and send an output to the neurons of the following layer. Eventually, the output neurons process the inputs received from the neurons of the last hidden layer and thus provide the ultimate prediction. The main hyperparameters tuned in order to minimize the error in the path from the input to the output predictions were: (a) *hidden_layer_size*, which indicates the number of neurons and the number of hidden layers; (b) *solver*, which is important to optimize the predictions at every decision step through the different layers; (c) *activation*, which refers to the activation function and defines how the weighted sum of the input is transformed into output by one or more nodes in a network layer; (d) *learning_rate_init*, which controls the step-size in updating the weights. In this work, three different architectures of hidden layers were tested, two of which presented three hidden layers formed by sets of [50, 50, 50] and [50, 100, 50] neurons, respectively, whereas the other tested architecture was formed by a single hidden layer of 100 neurons. As the type of solver, we tested *lbfgs*, which uses a limited amount of computer memory, only storing a certain amount of vectors, as well as the stochastic gradients *adam* and *sgd*. Among the activation functions, we considered “identity”, “logistic”, “tanh” and “relu” functions. The options investigated for *learning_rate_init* were 0.01, 0.001, 0.0001 and 0.00001.

### 3.4. Model Building and Evaluation

The 6 different molecular FPs and 4 different ML algorithms were combined for generating our models, resulting in a total of 24 possible combinations. An optimization process based on the Grid Search cross-validation implemented in Scikit-learn was applied to all generated models for determining the best hyperparameters setting. Grid Search is a generic approach provided in Scikit-learn for searching hyperparameters in order to maximize the performance of models. It employs a 5-fold stratified cross-validation (CV) to explore all possible combinations of hyperparameters, assigning a score to each of them. In this work, the scoring metric used was Matthew’s Correction Coefficient (see next section). After tuning the hyperparameters, a further 10-fold CV was performed for each model. A random training-test set splitting strategy was used, thus, for each fold, 30% of the starting dataset was considered as a test set. Finally, for each algorithm, the model with the fingerprint that obtained the highest average MCC was selected, resulting in 4 top-scored models considered for further analysis.

### 3.5. Model Evaluation

To check the performance of the 4 top-scored models selected, three statistical parameters were taken into account: Precision, Recall (or Sensitivity) and Matthew’s Correction Coefficient (MCC), which are defined as follows:

$$\mathrm{Precision}=\frac{\mathrm{TP}}{\left(\mathrm{TP}+\mathrm{FP}\right)}$$

$$\mathrm{Recall}=\frac{\mathrm{TP}}{\left(\mathrm{TP}+\mathrm{FN}\right)}$$

$$\mathrm{MCC}=\frac{\left(\mathrm{TP}\times \;\mathrm{TN}\;–\;\mathrm{FP}\times \mathrm{FN}\right)}{\sqrt{\left(\mathrm{TP}+\mathrm{FP}\right)\left(\mathrm{TP}+\mathrm{FN}\right)\left(\mathrm{TN}+\mathrm{FP}\right)\left(\mathrm{TN}+\mathrm{FN}\right)}}$$

TP (true positives) and TN (true negatives) correspond to the number of active and inactive compounds, respectively, correctly predicted as such; FP (false positives) represents the number of inactive compounds predicted as active, whereas FN (false negatives) represents the number of active compounds predicted as inactive. Precision is an index that assesses how much the model is able to provide correct positive predictions. In other words, it reports the ratio of the number of the correct positive predictions on the total positive predictions, therefore also including false positives. Recall indicates the number of active compounds correctly classified over the total number of actives. Both Precision and Recall return values in the range of 0–1, where 1 corresponds to the maximum model performance. MCC takes into account all values of the confusion matrix derived from the binary classification. It is a global index with values ranging from −1 to 1, where 1 corresponds to a perfect classification, 0 is a totally random classification and −1 indicates a complete inverse classification.

### 3.6. Consensus Approach

The consensus approach is based on the probability score (PS) provided by each model. These scores are calculated independently by each model and they are associated with the final predictions. Specifically, 0 ≤ PS < 0.5 corresponds to an inactive prediction, whereas 0.5 ≤ PS ≤ 1 corresponds to an active prediction. Moreover, the closer the score is to the extremes 0 and 1, the more confident is the prediction of inactivity and activity, respectively. The consensus strategy consists of combining the PSs generated for each molecule in the test set by the best models, as previously performed [32]. Specifically, based on the consensus approach, a compound was classified as active only if the calculated average of the PS provided by the best models resulted in at least 0.5. The evaluation of the consensus strategy was measured with the same statistical parameters used to evaluate the performance of the individual models (Precision, Recall and MCC). Therefore, performance indices were calculated based on the consensus predictions to evaluate the performance of such a strategy.

### 3.7. Database Generation and Machine Learning Screening

Approximately 70,000 total compounds from the commercial Enamine and Vitas-M databases were used as the screening database. In particular, specific sections of those databases, which are designed for the discovery of novel protein kinase inhibitors, were considered and subjected to the same data curation workflow preformed for training and test sets. The best developed model was used to screen the database in order to identify potential new active compounds. Only compounds that achieved the maximum probability score for each database were retrieved in this search.

### 3.8. In Vitro Cdk5 Inhibition Activity

Compounds identified by virtual screening were purchased from Enamine and Vitas-M and tested for Cdk5 inhibitory activity using the in vitro fluorescent bases assay e Z’-LYTE (Thermo Fisher Scientific, Madison, WI, USA). IC_50_ values were determined from logistical dose-response curves with 10 points, each obtained as the mean of two independent experiments. The development reaction interference and the compound fluorescence interference evaluation were carried out for each concentration of test compound assayed. The development reaction interference was established by comparing the test compound control wells that did not contain ATP versus the 0% phosphorylation control (which did not contain the test compound). The test compound fluorescence interference was determined by comparing the test compound control wells that did not contain the Kinase/Peptide Mixture (zero peptide control) versus the 0% inhibition control. Both compounds CPD1 and CPD4 were classified as non-interfering compounds. The structural identity of the purchased compounds was verified by ^1^H-NMR studies (Bruker Avance III 400 MHz spectrometer).

### 3.9. Molecular Docking Calculations

All docking calculations were carried out using the X-ray structure of Cdk5 in complex with a naphtyridine inhibitor (PDB code 7VDR) [34]. GOLD 5.1 with PLP fitness function was employed for molecular docking. The binding cavity used for the docking calculations was defined in order to include all residues, which stayed within 10 Å from the center of the co-crystallized ligand in the reference X-ray complex. In all docking studies performed, the possibility for the ligand to flip ring corners was enabled while the “allow early termination” option was deactivated. The ligands were subjected to 100 genetic algorithm runs and GOLD default values were used for all other settings [49]. The different docking poses relative to CPD1 and CPD4 were clustered with an RMSD cut-off of 2.0 Å. The representative poses of the obtained clusters with a population of at least 5% of the total solutions, which showed at least one H-bond with the hinge region of the protein, were selected as the most reliable poses for further analysis.

### 3.10. Molecular Dynamics (MD) Simulations

All simulations were performed using AMBER [50] version 20 and were carried out using the ff14SB force field at 300 K. General Amber force field (GAFF) parameters were assigned to the ligands, whereas partial charges were calculated using the AM1-BCC method with the Antechamber suite of AMBER 20. All ligand–protein complexes were placed in a rectangular parallelepiped water-box, by using a TIP3P explicit solvent model and solvated with a 15.0 Å water cap. Either Na+ or Cl− ions were added as counterions for the neutralization of the systems. Before MD simulations, two stages of energy minimization were carried out; in the first step, a position restraint of 100 kcal/(mol·Å^2^) was applied to the complex, thus minimizing only the position of the water molecules through 5000 steps of steepest descent, followed by conjugate gradient until a convergence of 0.05 kcal/(mol·Å^2^) was achieved. Successively, the whole system was energy minimized imposing a harmonic force constant of 10 kcal/(mol·Å^2^) only on the protein α carbons. The minimized complexes were used as starting conformations for the MD simulations. Periodic boundary conditions and particle mesh Ewald (PME) electrostatics were employed in the simulations. An initial MD step of 0.5 ns with constant-volume periodic boundary conditions was performed and the temperature of the system was raised from 0 to 300 K. The system was then equilibrated through 3 ns of constant pressure periodic boundary MD, employing the Langevin thermostat in order to keep the temperature of the system constant. Then, an additional 96.5 ns of constant pressure MD production was performed. Thus, a total of 100 ns of MD simulation was carried out for each protein–ligand complex analyzed in this study. All the α carbons of the protein were restrained with a harmonic force constant of 10 kcal/(mol·Å^2^) during the whole MD simulation. All the obtained MD trajectories were analyzed using the cpptraj program implemented in AMBER 20.

### 3.11. Binding Energy Evaluation

The evaluation of the binding energy associated with different ligand–protein complexes analyzed through MD simulations was carried out using AMBER 20, as already reported [51,52]. The trajectories relative to the last 50 ns of each simulation were extracted and used for the calculation for a total of 100 snapshots (at time intervals of 100 ps). Van der Waals, electrostatic and internal interactions were calculated with the SANDER module of AMBER 20, whereas polar energies were calculated using the Poisson−Boltzman methods with the MM-PBSA module of AMBER 20. Dielectric constants of 1 and 80 were used to represent the gas and water phases, respectively, whereas MOLSURF program was employed to estimate the nonpolar energies. The entropic term was considered as approximately constant in the comparison of the ligand−protein energetic interactions.

### 3.12. Cell Viability Assay

Human breast MDA-MB-231, colorectal HCT116 and ovarian OVCAR3 and A2780 cancer cells (from ATCC) were maintained at 37 °C in a humidified atmosphere containing 5% CO_2_ according to the supplier. Cells (5 × 10^2^) were plated in 96-well culture plates. The day after seeding, vehicles or compounds were added at different concentrations to the medium. Compounds were added to the cell culture at a concentration ranging from 200 to 0.02 μM. Cell viability was measured after 96 h according to the supplier (Promega, G7571) with a Tecan M1000 instrument. IC_50_ values were calculated from logistical dose response curves. Averages were obtained from triplicates, and error bars are standard deviations.

## 4. Conclusions

Cyclin-dependent kinase 5 (Cdk5) is considered as a promising target in the drug design field for its role in the progression of neurodegenerative diseases such as AD and PD, as well as in the development and progression of a variety of tumors. In this work, we employed a machine learning-based virtual screening protocol with the aim to identify novel potential Cdk5 inhibitors. Among 24 different machine learning models herein developed for this purpose, the best one in terms of MCC and Precision, was used to filter two focused libraries of commercial compounds. Experimental assays demonstrated a good Cdk5 inhibitory activity for two compounds, CPD1 and CPD4, out of the four ligands selected through the virtual screening, for a hit rate of 50%. Furthermore, CPD1 demonstrated a remarkable antiproliferative activity in ovarian and colon cancer cells. These findings validated the reliability of the in silico workflow and highlighted the identification of two promising hit compounds for the development of potent Cdk5 inhibitors. Moreover, the binding dispositions of these compounds into the Cdk5 active site were predicted through molecular docking, followed by MD simulations with binding free energy evaluations, thus providing a valuable starting point for structure-based hit-optimization studies.

## Figures and Tables

**Table 1:** Performance evaluation results, based on test set prediction, obtained with the 4 top-scored models and consensus strategy.

Model	MCC	Precision	Recall
Consensus	0.43	0.88	0.31
RF	0.42	0.87	0.30
SVM	0.42	0.65	0.52
MLP	0.41	0.59	0.61
KNN	0.33	0.58	0.46

**Table 2:** Structure and Cdk5 inhibition activity of the tested compounds.

Compound ID	Structure	IC_50_ (μM)
**CPD1**	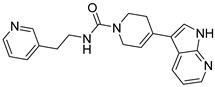	3.43 ± 0.01
**CPD2**	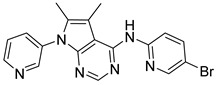	>30.0
**CPD3**	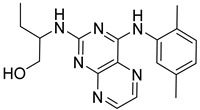	>30.0
**CPD4**	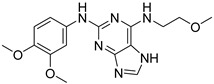	1.27 ± 0.07

**Table 3:** Cell growth inhibitory activities (IC_50_ values) of compounds CPD1 and CPD4.

IC_50_ (Mean ± SD, nM)				
Compound	HCT116	MDA-MB-231	OVCAR3	A2780
**CPD1**	183 ± 27	1474 ± 44	12.0 ± 1.1	93.7 ± 21.6
**CPD4**	1766 ± 230	2127 ± 349	2118 ± 63	678 ± 123
**Cisplatin**	2218 ± 6	>10,000	681 ± 50	275 ± 39

**Table 4:** MM-PBSA results for the analyzed Cdk5-CPD1/4 complexes. ΔPBSA is the sum of the van der Waals (VDW), electrostatic (ELE), as well as polar (EPB) and non-polar (ENPOLAR) solvation free energy. Data are expressed as kcal·mol^−1^.

CPD1	VDW	ELE	EPB	ENPOLAR	ΔPBSA
CL1	−47.2	−32.4	48.0	−4.3	−35.9
CL2	−47.0	−40.2	58.3	−4.2	−33.1
CL6	−46.8	−23.6	48.6	−4.3	−26.0
CL4	−44.6	−23.6	49.2	−4.2	−23.2
**CPD4**	**VDW**	**ELE**	**EPB**	**ENPOLAR**	**ΔPBSA**
CL7	−45.6	−24.8	40.2	−4.2	−34.4
CL1	−44.7	−24.1	43.8	−4.4	−29.4
CL2	−44.9	−23.2	48.2	−4.3	−24.3
CL9	−37.2	−22.6	44.4	−4.1	−19.5

## Data Availability

All compounds included in the dataset used for model building and evaluation were downloaded from ChEMBL 25 (https://www.ebi.ac.uk/chembl/ accessed on 1 June 2022). Information about the hyperparameters settings evaluated during the optimization process of each model is reported within the Materials and Methods section of the manuscript. The average MCC values obtained for the 24 models developed, as result of the cross validation, are reported within the Supporting Information (Tables S1–S4). The detailed hyperparameters setup of the best performing model, employed for the virtual screening study, is reported in the Supporting Information (Table S5). Such a model is freely accessible as a web tool at the following page: http://www.mmvsl.it/wp/cdk5-predictor/. The two libraries of commercial compounds screened by the model were downloaded from Enamine (https://enamine.net/ accessed on 1 June 2022) and Vitas-M (https://vitasmlab.biz/ accessed on 1 June 2022) laboratories.

## Supplementary Materials

The following supporting information can be downloaded at: https://www.mdpi.com/article/10.3390/ijms231810653/s1.
