# Supplementary material for: Machine Learning-Based Virtual Screening for the Identification of Cdk5 Inhibitors
Source: Int J Mol Sci. 2022 Sep 13;23(18):10653. doi: 10.3390/ijms231810653 (PMC9502400; doi:10.3390/ijms231810653)
Supplement: Supplementary file 1 [file ijms-23-10653-s001.zip › ijms-1910729-supplementary.pdf]

# Supporting Information

## Machine learning-based virtual screening for the identification of Cdk5 inhibitors

*Miriana Di Stefano[a,b], Salvatore Galati[a], Gabriella Ortore[a], Isabella Caligiuri[c], Flavio Rizzolio[c,d], Costanza Ceni[a,b], Simone Bertini[a], Giulia Bononi[a], Carlotta Granchi[a], Marco Macchia[a], Giulio Poli[a,*], Tiziano Tuccinardi[a]*

[a] Department of Pharmacy, University of Pisa, Via Bonanno 6, 56126 Pisa, Italy.

[b] Department of Life Sciences, University of Siena, Via Aldo Moro, 2, 53100 Siena, Italy.

[c] Pathology Unit, Centro di Riferimento Oncologico di Aviano (CRO) IRCCS, 33081 Aviano, Italy.

[d] Department of Molecular Sciences and Nanosystems, Ca' Foscari University, 30123 Venezia, Italy.

### Table of Contents

**Table S1.** Cross validation results obtained for RF-based models. S2

**Table S2.** Cross validation results obtained for KNN-based models. S2

**Table S3.** Cross validation results obtained for SVM-based models. S3

**Table S4.** Cross validation results obtained for MLP-based models. S3

**Table S5.** Hyperparameters setup of the best performing model, used for virtual screening. S4

**Figure S1.** CDK5 $IC_{50}$ plots for compounds CPD1 and CPD4. S4

**Table S1.** Evaluation results, expressed in terms of MCC, obtained for RF-based models developed through cross-validation analysis.

| Fingerprint | MCC |
|---|---|
| Morgan | 0.65 ± 0.05 |
| Layer | 0.63 ± 0.05 |
| Pattern | 0.62 ± 0.06 |
| Pharm2D | 0.60 ± 0.05 |
| RDKit | 0.59 ± 0.05 |
| MACCS | 0.54 ± 0.05 |

**Table S2.** Evaluation results, expressed in terms of MCC, obtained for KNN-based models developed through cross-validation analysis.

| Fingerprint | MCC |
|---|---|
| Morgan | 0.60 ± 0.04 |
| RDKit | 0.59 ± 0.05 |
| Layer | 0.59 ± 0.03 |
| Pharm2D | 0.54 ± 0.06 |
| Pattern | 0.53 ± 0.05 |
| MACCS | 0.50 ± 0.06 |

**Table S3.** Evaluation results, expressed in terms of MCC, obtained for SVM-based models developed through cross-validation analysis.

| **Fingerprint** | **MCC** |
|---|---|
| Morgan | 0.69 ± 0.04 |
| Pattern | 0.63 ± 0.05 |
| RDKit | 0.60 ± 0.08 |
| Pharm2D | 0.60 ± 0.03 |
| Layer | 0.59 ± 0.07 |
| MACCS | 0.55 ± 0.07 |

**Table S4.** Evaluation results, expressed in terms of MCC, obtained for MLP-based models developed through cross-validation analysis.

| **Fingerprint** | **MCC** |
|---|---|
| Morgan | 0.67 ± 0.08 |
| Pattern | 0.59 ± 0.05 |
| RDKit | 0.58 ± 0.11 |
| Pharm2D | 0.58 ± 0.05 |
| Layer | 0.53 ± 0.05 |
| MACCS | 0.47 ± 0.06 |

**Table S5.** Hyperparameters setup of the best performing model, used for virtual screening.

| Hyperparameter | Value |
|---|---|
| Max_features | log2 |
| n_estimators | 500 |
| Class_weight | None |
| Min_samples_split | 2 |
| Min_samples_leaf | 1 |
| Max_depth | None |

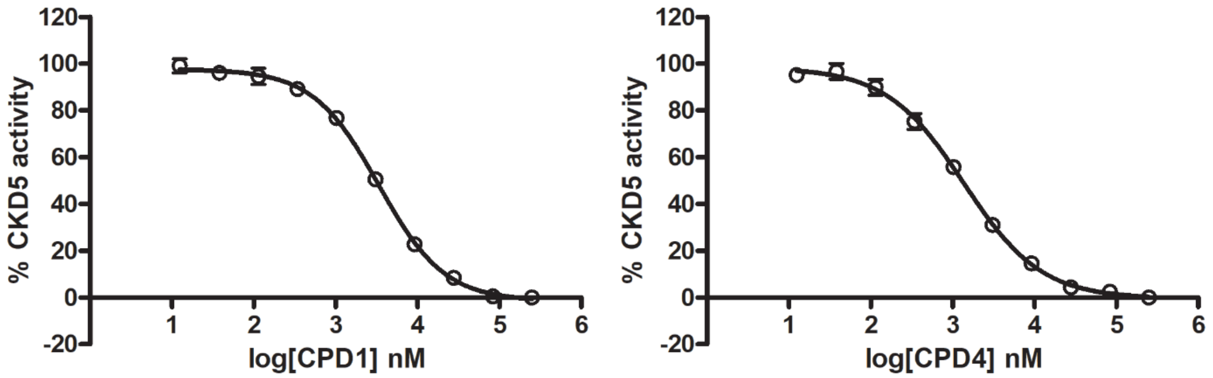


**Figure S1.** CDK5 $IC_{50}$ plots for compounds **CPD1** (left side) and **CPD4** (right side).
